# PKAN neurodegeneration and residual PANK2 activities in patient erythrocytes

**DOI:** 10.1002/acn3.51127

**Published:** 2020-07-23

**Authors:** Maike Werning, Ernst W. Müllner, Georg Mlynek, Verena Dobretzberger, Kristina Djinovic‐Carugo, David M. Baron, Holger Prokisch, Boriana Büchner, Thomas Klopstock, Ulrich Salzer

**Affiliations:** ^1^ Center for Medical Biochemistry Max Perutz Labs Medical University of Vienna Vienna Austria; ^2^ Department of Structural and Computational Biology Max Perutz Labs University of Vienna Vienna Austria; ^3^ Department of Biochemistry Faculty of Chemistry and Chemical Technology University of Ljubljana Ljubljana Slovenia; ^4^ Department of Anaesthesia Intensive Care Medicine and Pain Medicine Medical University of Vienna Vienna Austria; ^5^ Institute of Human Genetics Technical University of Munich Munich Germany; ^6^ Institute of Human Genetics Helmholtz Center Munich German Research Center for Environmental Health Neuherberg Germany; ^7^ Department of Neurology Friedrich‐Baur‐Institute University Hospital LMU Munich Munich Germany; ^8^ German Center for Neurodegenerative Diseases (DZNE) Munich Germany; ^9^ Munich Cluster for Systems Neurology (SyNergy) Munich Germany

## Abstract

**Objective:**

Pantothenate kinase 2‐associated neurodegeneration (PKAN) is a rare neurodegenerative disease caused by mutations in the pantothenate kinase 2 (*PANK2*) gene. PKAN is associated with iron deposition in the basal ganglia and, occasionally, with the occurrence of misshaped erythrocytes (acanthocytes). The aim of this study was to assess residual activity of PANK2 in erythrocytes of PKAN patients and to correlate these data with the type of *PANK2* mutations and the progression of neurodegeneration.

**Methods:**

Residual PANK2 activities in erythrocytes of 14 PKAN patients and 14 related carriers were assessed by a radiometric assay. Clinical data on neurodegeneration included the Barry–Albright Dystonia Scale (BAD‐Scale) besides further general patient features. A molecular visualization and analysis program was used to rationalize the influence of the PKAN causing mutations on a molecular level.

**Results:**

Erythrocytes of PKAN patients had markedly reduced or no PANK2 activity. However, patients with at least one allele of the c.1583C > T (T528M) or the c.833G > T (R278L) variant exhibited 12–56% of residual PANK2 activity. In line, molecular modeling indicated only minor effects on enzyme structure for these point mutations. On average, these patients with c.1583C > T or c.833G > T variant had lower BAD scores corresponding to lower symptom severity than patients with other *PANK2* point mutations.

**Interpretation:**

Residual erythrocyte PANK2 activity could be a predictor for the progression of neurodegeneration in PKAN patients. Erythrocytes are an interesting patient‐derived cell system with still underestimated diagnostic potential.

## Introduction

Pantothenate kinase 2‐associated neurodegeneration (PKAN) is a neurodegenerative disease caused by mutations in the pantothenate kinase 2 gene (*PANK2*),^1^ and represents the most common genetic form of NBIA (neurodegeneration with brain iron accumulation). PKAN is characterized by progressive generalized dystonia, and imaging shows excess iron deposition in the basal ganglia, specifically the globus pallidus and the substantia nigra. Patients with an early onset before 6 years of age have been historically classified as classic PKAN (cPKAN), resulting in more severe disease. Atypical PKAN (aPKAN) has a later onset (>10 years) and progresses more slowly.^2^

In some PKAN patients, misshaped erythrocytes with thorny protrusions (so‐called “acanthocytes”) are found in blood. This establishes a link to the neuroacanthocytoses (NA) syndromes,^3^ such as McLeod syndrome, chorea‐acanthocytosis, and Huntington's disease‐like 2, which are also characterized by movement disorders due to neurodegeneration in the basal ganglia. While these acanthocytes are clinically irrelevant (e.g., not associated with anemia), they constitute an interesting cellular model system to study molecular mechanisms of the disease.^4^, ^5^, ^6^, ^7^, ^8^, ^9^

PANK2 encoded by the *PANK2* gene is one of four isoforms of pantothenate kinases (PANK1‐4) catalyzing the rate‐limiting step in coenzyme A (CoA) biosynthesis. PANK2 has a mitochondrial targeting sequence and in neurons is localized to mitochondria.^10^, ^11^ More recently, both a nuclear localization and a nuclear export signal were also identified and complex subcellular targeting of PANK2 was reported.^12^ Human PANK2 is inhibited by CoA and various acyl‐CoA metabolites with IC_50_ values in the high nanomolar range^13^ and activated by palmitoylcarnitine at low micromolar concentrations^14^ indicating that the fine‐tuning of its activity is crucial for the regulation of the cellular lipid metabolism. PANK1 and PANK3 are assumed to be localized in the cytosol and their activities are distinctly affected by CoA and acyl‐CoA metabolites.^15^

A large variety of mutations have been identified in *PANK2* in PKAN patients.^16^, ^17^ Interestingly, a considerable fraction of these are point mutations indicating that single amino acid substitutions might critically affect the function of PANK2 in the cellular context. Biochemical studies on recombinant wild type (WT) and mutant PANK2 revealed that only few of the tested point mutations strongly affected pantothenate kinase activity.^13^ Some exhibited only mildly reduced and some even elevated activity as compared to WT PANK2. Similar results were obtained when PANK3 proteins with mutations corresponding to respective PKAN‐associated *PANK2* mutations were analyzed.^18^ These studies suggest that some deleterious effects of point mutations only develop in the specific cellular context and may not become evident when respective mutant proteins are overexpressed in cell culture systems.

In this study we characterized endogenous mutant PANK2 protein in erythrocytes from 14 PKAN patients, assessed their residual PANK2 activity in the erythrocyte cytosol and correlated these with molecular models of the point mutations and clinical data of the patients.

## Materials and Methods

### Blood sample collection

Blood was collected within the Global NBIA Biobank (Technical University of Munich) and/or from the Muscle Tissue Culture Collection (Friedrich‐Baur‐Institute, Klinikum of Ludwig‐Maximilians‐University of Munich). Informed consent was obtained from each participant (IRB‐ID: TUM 5578‐12s or KUM 45‐14). This study was also approved by the ethics committee of the Medical University of Vienna (EK‐Nr. 1148/2015). Nine milliliter of blood from 14 patients, 14 carriers and a healthy control (HC1) in Munich were collected by venipuncture into EDTA vacutainers and sent to Vienna overnight at 4–8°C. There, freshly drawn blood from local donors (HC2‐6) was used to control transport‐induced alterations.

### Genetic and clinical evaluation of the patients

Genetic and clinical data have been collected within the Global NBIA Registry (IRB‐ID 187‐12) and shared for the purposes of this study in an anonymized way (Table 1). Data shared included the Barry–Albright Dystonia (BAD) score, a scale to rate severity of dystonia in eight different body regions (eyes, mouth, neck, trunk, both arms, and both legs) with total scores ranging from 0 (no dystonia) to 32 (severe generalized dystonia). Within the Global NBIA registry, only the threshold of 6 years of age at the onset of motor symptoms has been chosen as definition for discrimination between cPKAN and aPKAN, without taking into account any other phenotypic features of the patients (e.g., disease progression). Thus, the Registry clinical diagnosis does not reflect the course of the disease. The variable BAD score / yr was calculated (BAD score divided by the age in years) to provide a rough measure for the progression of neurodegeneration.

**Table 1:** Clinical and genetic data of PKAN patients in this study.

Patient	Gender	Age	Age of onset	Diagnosis	BAD score	Age at BAD‐score	BAD/age	DNA mutation		Protein mutation	
								Allele 1	Allele 2	Allele 1	Allele 2
P1	**♂**	21	2	classical	29	21	1,38	c.573del	c.1274T > C	S191fs	L425P
P2	♀	15	23	atypical	14	27	0,52	c.573del	c.863C > G	S191fs	P288R
P3	♂	25	1	classical	13	9	1,44	c.1561G > A	c.1561G > A	G521R	G521R
P4	♀	28	10	atypical	29	19	1,53	c.1660G > A	c.1660G > A	E554K	E554K
P5	♀	13	12	atypical	16	34	0,47	c.1369G > T	c.1561G > A	D457Y	G521R
P6	**♂**	23	3	classical	31	23	1,35	c.1660G > A	c.1660G > A	E554K	E554K
P7	♀	27	14	atypical	11	20	0,55	c.573del	c.863C > G	S191fs	P288R
P8	♀	22	11	atypical	11	13	0,85	c.1420‐1426del	c.1583C > T	N474fs	T528M
P9	♂	25	13	atypical	22	22	1,00	c.1561G > A	c.1583C > T	G521R	T528M
P10	**♂**	25	12	atypical	20	21	0,95	c.1561G > A	c.1583C > T	G521R	T528M
P11	♀	38	10	atypical	13	25	0,52	c.1583C > T	c.1235 + 5G>A	T528M	**?**
P12	**♂**	36	2	classical	9	11	0,82	c.833G > T	c.833G > T	R278L	R278L
P13	**♂**	11	2	classical	9	36	0,25	c.1583C > T	c.1583C > T	T528M	T528M
P14	♂	25	16	atypical	12	23	0,52	c.1583C > T	c.1583C > T	T528M	T528M

The DNA mutations refer to the NCBI Reference Sequence: NM_153638.3 (pantothenate kinase 2 mRNA transcript variant 1). The c.1235 + 5G > A mutation of patient P11 is located in the intron 5 bases downstream the splice site after exon 3 and changes the consensus sequence from GTAAGT to GTAAAT which likely results in improper splicing.

### Pantothenate kinase (PANK) activity assay

PANK activity was determined as described^19^ with modifications. Washed erythrocytes were hypotonically lysed, centrifuged (16,000*g*, 20 min, 4°C), and total protein in the supernatant was determined at 280 nm (NanoDrop 2000C, ThermoFisherScientific). Two hundred and fifty microgram of extract were mixed with 11.25 *µ*mol/L ^14^C‐labeled D‐pantothenic acid (50 mCi/mmol; American Radiolabeled Chemicals), 2.5 mmol/L ATP, 10 mmol/L MgCl_2_ and 0.1 mol/L Tris/Cl pH = 7.5 in 40 *µ*L and incubated at 37°C. The reaction mixture was transferred onto an ion‐exchange filter (GE Whatman) and immersed in 95% ethanol/1% acetic acid. The filter was suspended in 3 mL scintillation liquid (PerkinElmer) and the radioactivity quantified in a scintillation counter (TRI‐CARB 2100TR, PerkinElmer).

### Immunoblot analysis

Hemoglobin was depleted from erythrocyte cytosol by Nickel Sepharose (GE Healthcare) and concentrated by ultrafiltration (cut‐off 30 kDa, Ultra‐15 Centrifugal Filter, Merck Millipore). Protein samples were separated by SDS‐PAGE and transferred to nitrocellulose (GE Healthcare). Membranes were blocked with 5% milk in TBS, incubated at 4°C with anti‐PANK2 antibody (sc‐82288, Santa Cruz) and at RT goat anti‐rabbit IgG, peroxidase‐conjugated antibody (Jackson ImmunoResearch). The chemiluminescence reaction (Pierce ECL Western Blotting Substrate; ThermoFisherScientific) was quantified in a ChemiDoc system (Bio‐Rad Laboratories). Carbonic anhydrase (CA, rabbit monoclonal anti‐carbonic anhydrase 1/CA1 antibody (ab108367, Abcam)) was used as a loading control.

### Molecular visualization

Structures of PANK1β in complex with acetyl‐CoA (PDB 2I7N), PANK3 bound to acetyl‐CoA (PDB 2I7P, 3MK6), to palmitoyl‐CoA (PDB 5KQD, PANK3·ATP·Mg^2+^ (PDB 5KPT), PANK3·AMPPNP·pantothenate (PDB 5KPR), PANK3·ADP·phosphopantothenate (PDB 5KPZ), PANK3·AMPPN (PDB 5KQ8), and PANK2·acetyl‐CoA (PDB 2I7N) were from the protein data bank. The three types of pantothenate kinases PANK1, PANK2, PANK3 have high sequence similarity (~83%) in the catalytic core and the crystal structures superpose well suggesting similar catalytic mechanisms.^20^ Figures of PANK2 mutations were created using pymol (https://pymol.org/2/).

### Statistical analyses

Data were analyzed with SPSS (version 25) (IBM) and GraphPad Prism (version 6) (GraphPad Software) using methods of descriptive statistics.

## Results

**Figure 1:**
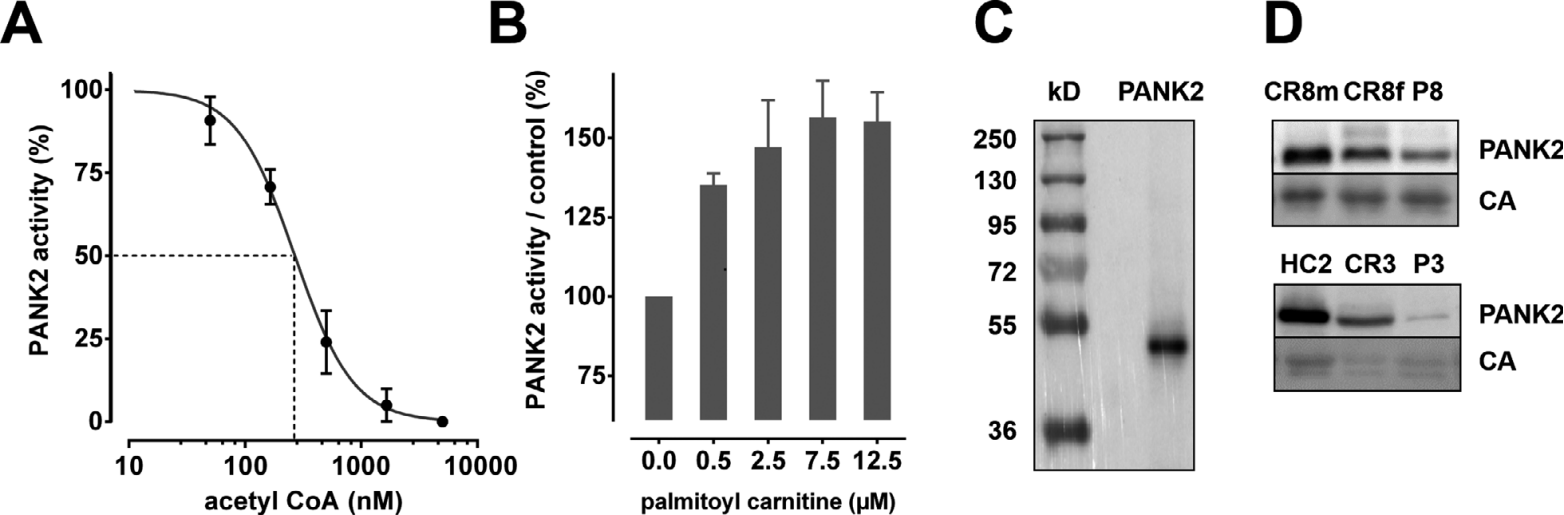
Pantothenate kinase activity and the presence of PANK2 protein in human erythrocytes. (A and B) Extracts of erythrocyte cytosol of healthy volunteers were tested for pantothenate kinase activity (see Materials and Methods) in the presence of the feedback inhibitor acetyl‐CoA (A) or the activator palmitoylcarnitine (B). The concentrations of acetyl‐CoA used are shown in a logarithmic scale. Each experiment was performed with blood from five healthy donors and each data set normalized to the control incubation without effector. Mean values and standard deviations are given in %. (C) A hemoglobin‐depleted erythrocyte cytosolic extract (see Materials and Methods) of a healthy donor was resolved by SDS‐PAGE and analyzed by Western blotting for PANK2 revealing a band of 49 kDa that conforms to the size of PANK2 in other cells types. (D) Cytosolic extracts of selected WT donors, patients and carriers were analyzed by Western blotting for PANK2 and carbonic anhydrase (CA as loading control) with bands at 49 and 29 kD, respectively. Note the considerably higher abundance of erythrocyte PANK2 in patient P8 (upper panel) as compared to patient P3 (lower panel).

PANK activity could be measured in crude cytosolic preparations of erythrocytes thereby confirming earlier data.^21^ Since the activities of the PANK isoforms are feedback inhibited by CoA and some of its metabolites^13^, ^15^ we tested the effect of acetyl‐CoA on erythrocyte pantothenate kinase activity. There was a dose‐dependent inhibition of PANK activity, with an IC_50_ of 0.26*µ*M acetyl‐CoA (Fig. 1A). This conforms well to the IC_50_ of PANK2^13^ and is clearly below that of PANK3 and PANK1β (1 and 5* µ*mol/L, respectively).^15^ Moreover, palmitoyl‐L‐carnitine increased erythrocyte PANK activity to the same extent and concentration range as reported for PANK2^14^ (Fig. 1B). Furthermore, a hemoglobin‐depleted extract of the erythrocyte cytosol was prepared and subjected to Western analysis. A polyclonal PANK2 antibody revealed a specific band of about 49 kD (Fig. 1C) which is in accordance with the size of the mature PANK2 protein.^11^ This band was (strongly) reduced in extracts of PKAN patients (Fig. 1D). In line with these data, the presence of PANK2 and the absence of other PANK isoforms in erythrocytes were found by proteomic analyses.^22^, ^23^

**Figure 2:**
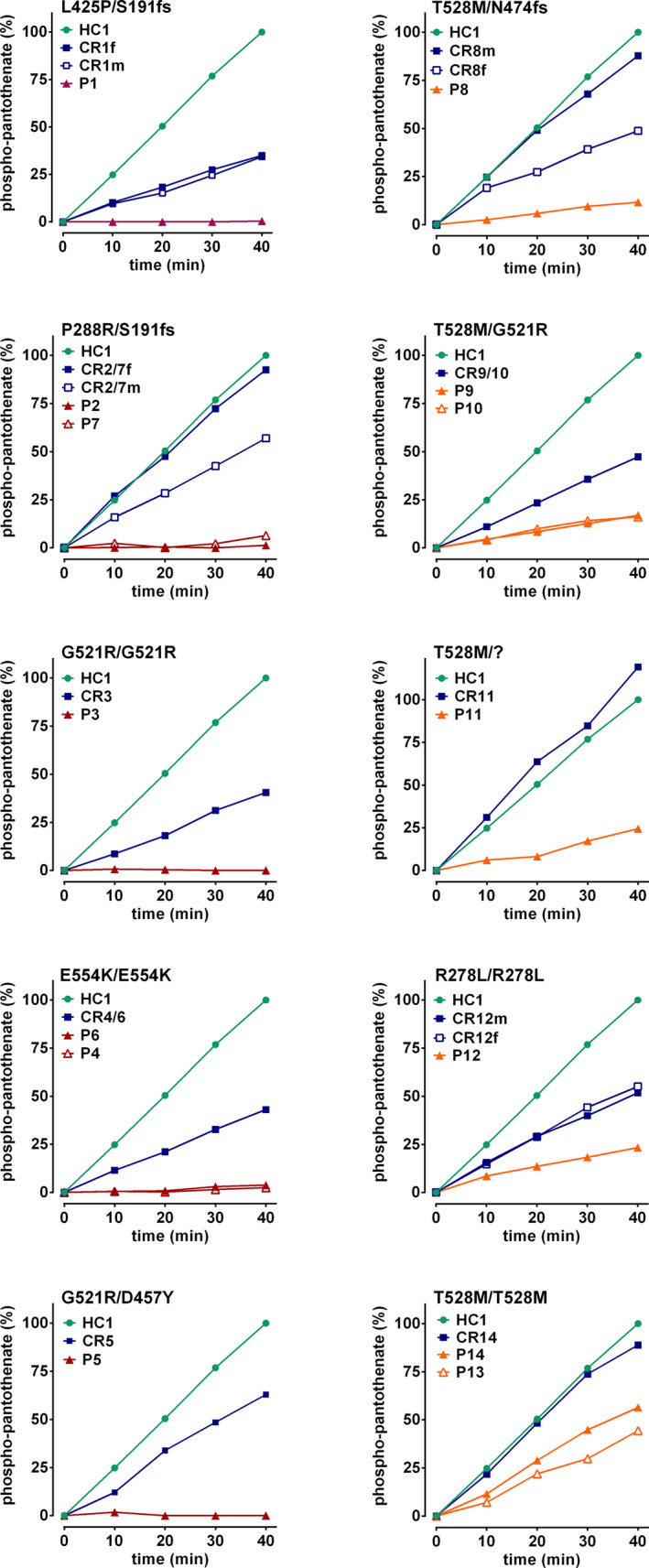
Phosphopantothenate generation is strongly reduced in the erythrocyte cytosol of PKAN patients. Generation of radiolabeled phosphopantothenate in erythrocyte cytosolic extracts was assessed as described in Materials and Methods. The relative amounts of phosphopantothenate were determined by normalization to the amount generated within 40 min in the extract of HC1 (which was set to 100%) and respective time courses are given. Patients with identical mutations are shown in the same panels together with respective carriers as indicated.

**Figure 3:**
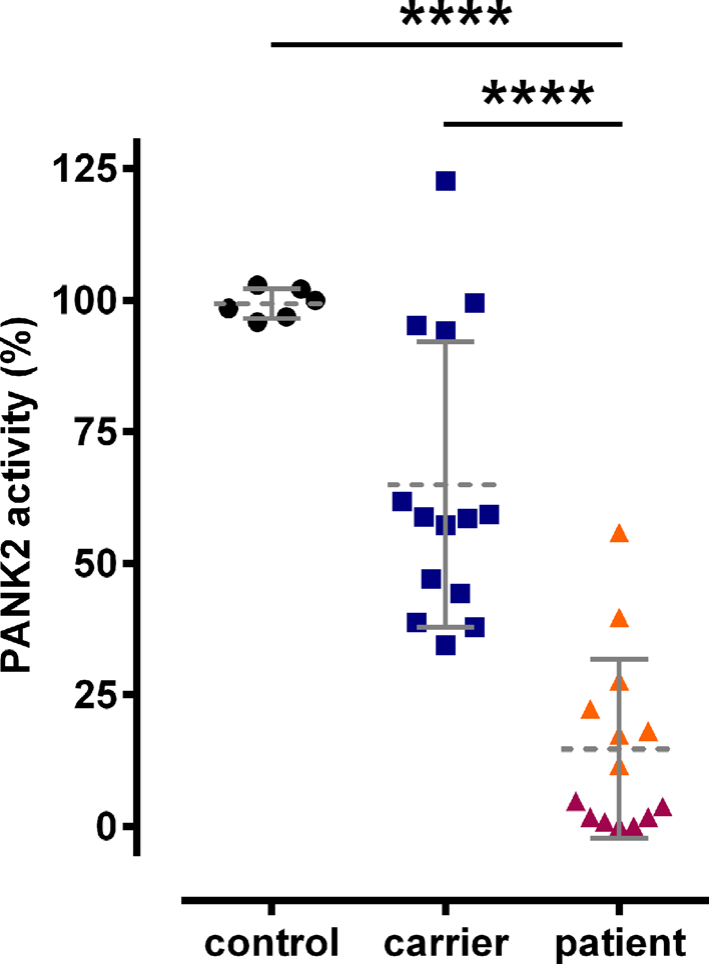
Comparison of erythrocyte PANK2 activity between control donors, carriers, and patients. Erythrocyte PANK2 activities are plotted for control donors (black circles), carriers (blue squares), and patients (orange and red triangles with or without at least one allele harboring a T528M or R278L, respectively). For numerical data, compare to Table 2. Statistical analyses (unpaired student’s t‐test) revealed the significance of the difference between the mean values of the groups (**** <0.001) as indicated.

We analyzed erythrocyte PANK activity of 14 PKAN patients, 14 related healthy carriers (father and/or mother of patients) and 6 healthy control donors. Healthy control donor HC1 donated blood at the same date and place (Munich) as the patients. Intra‐donor variation of PANK activity of the HC1 samples was within the range of intra‐ and inter‐donor variation of freshly drawn samples from healthy donors (HC2‐HC6) in Vienna where the assays were performed indicating that sample shipment did not affect PANK activity. HC1 therefore served as reference for normalization. Patients P1 to P7 had very low or absent PANK activities (<5% of control). This again strongly indicates that PANK2 is the only PANK isoform in human erythrocytes. In contrast, patients P8 to P14 had considerable residual activities (>10% of control); P13 and P14 had 40% and 56% of control, respectively (Fig. 2 and Table 2). Six of the patients with PANK activities >10% were homo‐ or heterozygous for the T528M mutation and P12 was homozygous for the R278L mutation. Carrier blood samples showed reduced PANK activity (except CR11). Expectedly, their PANK activities were higher than those of the related patients (Fig. 2 (families are grouped in panels) and Fig. 3).

**Table 2:** Relative PANK2 activity in the erythrocyte cytosol.

ID	Activity (%)	StDev (%)	*n*
HC1	100.00	2.30	36
HC2	102.13	8.27	36
HC3	97.13	4.93	9
HC4	95.20	0.58	9
HC5	106.26	14.03	9
HC6	91.92	13.92	9
CR1f	37.83	2.24	4
CR1m	34.44	2.92	4
CR2/7f	99.41	5.87	4
CR2/7m	59.34	2.93	4
CR3	38.78	2.90	4
CR4/6	44.32	1.34	4
CR5	61.84	7.43	4
CR8f	95.21	4.77	8
CR8m	58.87	10.83	8
CR9/10	47.03	1.45	4
CR11	122.73	5.48	4
CR12f	57.20	4.15	4
CR12m	58.55	1.55	4
CR14	94.16	4.86	4
P1	0.10	0.18	4
P2	0.11	0.20	4
P3	0.91	1.14	8
P4	1.81	0.82	8
P5	1.82	3.10	4
P6	3.81	0.95	8
P7	4.84	3.47	4
P8	11.67	0.87	8
P9	17.42	0.52	4
P10	18.18	1.52	4
P11	22.38	3.35	4
P12	27.70	4.29	12
P13	39.76	6.44	4
P14	55.87	5.26	4

Erythrocyte extracts of healthy control donors (HC), carriers (CR) and PKAN patients (P) were assayed for phospho‐pantothenate generation as described in the Materials and Methods section and the specific PANK2 activities per total protein were determined. Relative PANK2 activities were calculated from the specific activities by normalization to the specific activity of donor HC1 that served as WT control in each experiment (activity denotes mean values of relative PANK2 activity in %; StDev denotes standard deviation; n denotes the number of data points).

**Figure 4:**
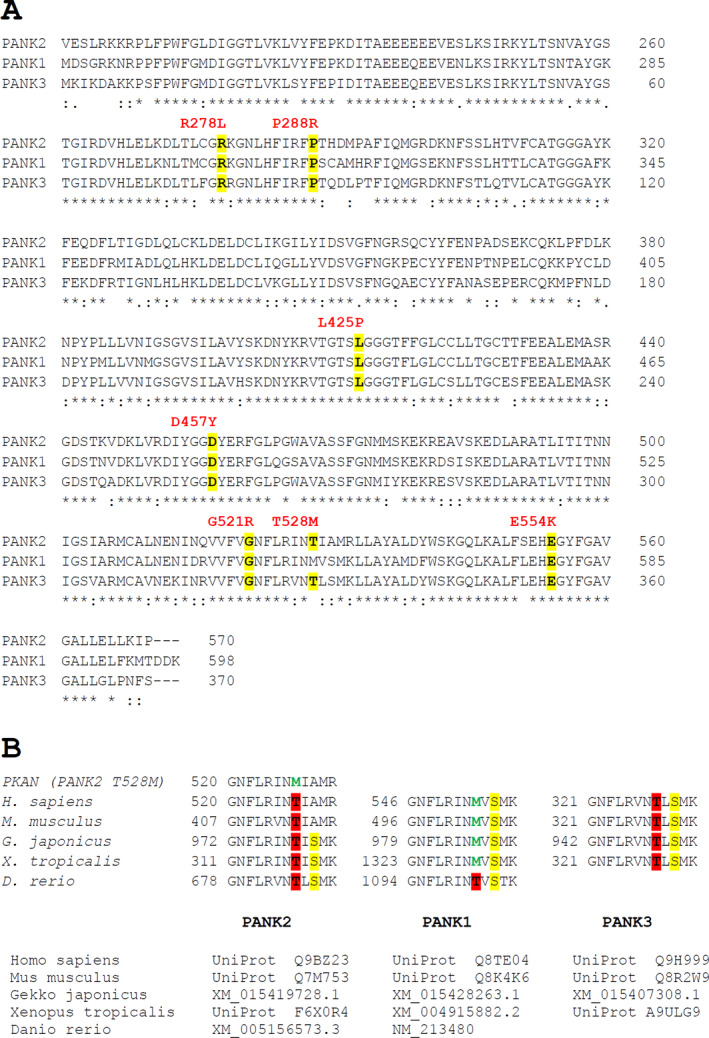
Conservation of PANK2 point mutations sites between the isoforms and phylogenetic variation within a partial sequence corresponding to the region around T528 of human PANK2. (A) Sequence alignment of the three human PANK isoforms was performed with the CLUSTALW algorithm using the Uniprot sequences of human PANK2 (Q9BZ23), PANK1 (Q8TE04), and PANK3 (Q9H999) with * (asterisk) and: (colon) indicating full conservation and conservation between groups of amino acids with highly similar properties, respectively. The seven PANK2 point mutations relevant for this study are indicated above the respective positions in red and amino acid conservation is highlighted in yellow. (B) Phylogenetic comparisons of PANK2 sequences around T528 between two mammalian, one reptile, one amphibian, and one fish species (species names and sequence identifiers below the alignments) are shown for the three PANK isoforms as indicated. Please note that the respective threonine corresponding to T528 of human PANK2 (highlighted in red) is conserved in PANK2 and PANK3 isoforms while it shifted to methionine (green) early in the evolution of the PANK1 protein. Conversely, the alanine at position 530 of human PANK2 is only found in mammalian PANK2 isoforms while phylogenetically distant species have a serine at this position (highlighted in yellow), which is also conserved in PANK1 and PANK3 isoforms.

**Figure 5:**
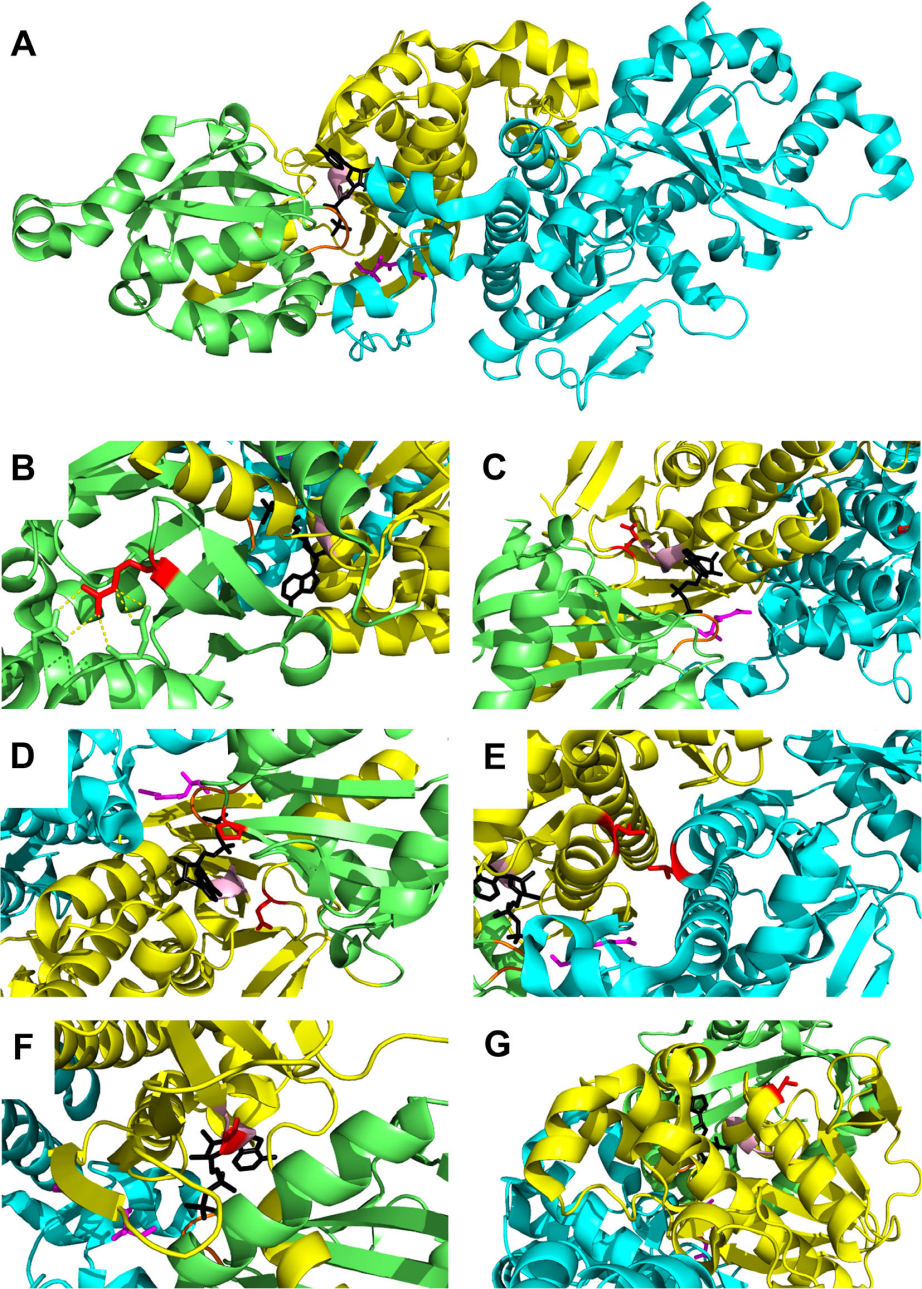
3D structure of human PANK2 and zoom‐in on point mutation sites. (A) PANK2 dimer structure (PDB 5e26) is shown in cartoon representation. Domain A of chain A is shown in lime, domain B of chain A in yellow, and chain B in cyan. Pantothenate is shown as pink sticks and adenosine diphosphate as black sticks. The glycine‐rich ATP binding loop is highlighted in orange and the short ATP binding helix in light pink. (B) Residue R278: shown as red stick. Yellow dashes indicate ionic and hydrogen bonding. (C) Residue D457: This amino acid is located in domain B, in a loop involved in pantothenate binding. (D) Residue P288 and E554: P288 maps to the interior of domain A, E554 is localized in domain B, in the vicinity of the ATP binding site. (E) Amino acid L425: This residue is situated on a helix next to the dimerization interface. (F) Amino acid G521 is located on the short ATP binding helix (G) Residue T528 in domain B is exposed to solvent with no other apparent interactions.

Each patient had at least one mutant allele resulting in a single amino acid exchange in the PANK2 sequence (Fig. 4A). To rationalize the considerable variation in residual PANK activity in patients, structural models of the point mutants were analyzed. PANK2 is a homodimer with each monomer consisting of two conserved domains, A (residues 208–356) and B (residues 357–566), and a 200 amino acid long N‐terminal region. Dimerization is mediated by long α‐helices (residues 486–513) and two reciprocal interactions between an extended loop (residues 446–471) and another helix (residues 414–426). The ATP binding site is located between domain A (residues 219–221) and domain B (residues 520–522) with the active site (E338) being located in domain A (Fig. 5A).

*Mutation R278L (patient P12)*: Residue R278 is at the surface of domain A and forms a salt bridge with E237 (Fig. 5B). Interestingly, the recombinant PANK3 mutant R78 corresponding to the PANK2 R278L mutant exhibited increased activity compared to WT PANK3. Erythrocytes of patient P12 (homozygous for R278M) have considerable PANK2 activity (27.7%).

*Mutation D457Y (patient P5)*: D457Y is in a pantothenate‐binding loop (domain B) and likely affects binding of the substrate (Fig. 5C).

*Mutations P288R and E554K (patients P2, P7, P4, and P6)*: P288 is in the interior of domain A next to the ATP binding groove, E554 is near the ATP binding site in domain B. Both mutations probably impair ATP binding (Fig. 5D).

*Mutation L425P (patient P1)*: Residue L425 is mapped on a helix at the dimerization interface. PANK proteins are highly cooperative allosteric enzymes exploiting structural changes at the dimer interface to coordinately switch between active and inactive conformations.^20^ Since proline is a potent helixbreaker, the L425P mutant likely exhibits an impaired activation–inactivation cycle (Fig. 5E).

*Mutation G521R (patients P9, P10, P5, and P3)*: G521 is located in the short ATP binding helix, probably impairing ATP binding (Fig. 5F). Recombinant G521R PANK2 mutant had no activity.^13^ In line, erythrocytes of P3 (homozygous for G521R) exhibit virtually no PANK activity (Fig. 2).

*Mutation T528M (P8, P9, P11, P10, P14, and P13)*: T528 is located on the surface of domain B with the amino acid pointing toward the solvent (Fig. 5G). Interestingly, both recombinant mutants of PANK2 (T528M) and PANK3 (T328M corresponding to the PANK2 T528M mutant) had increased activity compared to wild‐type PANK2.^13^, ^20^ Conversely, functional testing of PANK2 mutants in an E. coli complementation assay revealed that the clone transformed with the p.T528M allele grew significantly better than any other tested mutant, but less well than the clone with the wild‐type allele.^17^ Erythrocytes of patients P13 and P14 (both homozygous for the T528M mutation) had residual PANK2 of 39.8% and 55.9%, respectively (Fig. 2, Table 2). Interestingly, the amino acid of PANK1 corresponding to T528 of PANK2 is actually a methionine (M553) (Fig. 4). Phylogenetic comparison of the sequences around this site shows a nearby serine conserved in all PANK isoforms and in all species except for the mammalian PANK2. The T528M mutation in *PANK2* therefore abrogates the only potential phosphorylation site in this region (Fig. 4B). Hence, hypothetically, the detrimental effect of this mutation is due to aberrant posttranscriptional regulation rather than to instability of the mutant protein.

**Figure 6:**
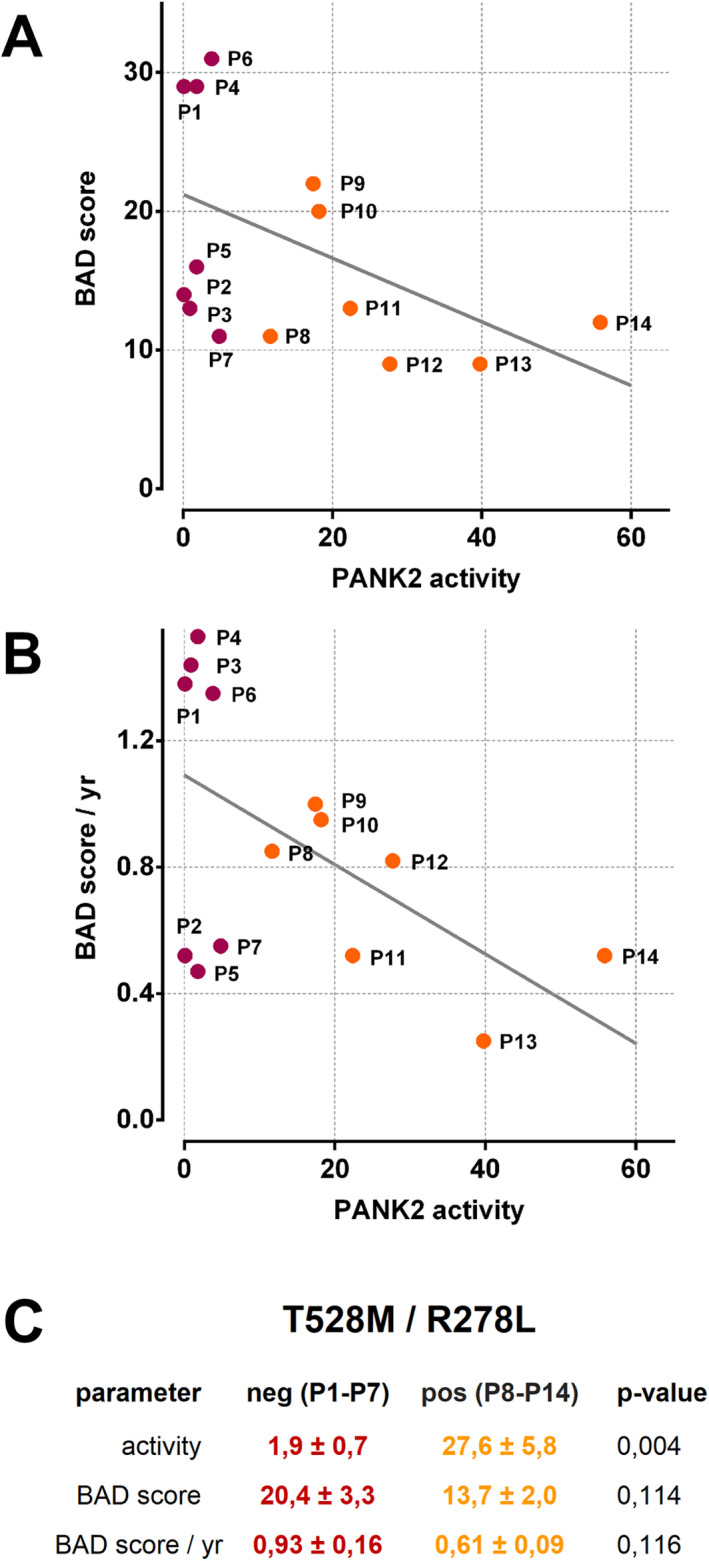
Negative correlation between residual erythrocyte PANK2 activity in patients and their score of neurodegeneration. Residual erythrocyte PANK2 activity is plotted against the BAD score (A) or an age‐normalized version thereof (B). Data points are identified by the patient IDs and either designated by orange circles (patients with at least one allele harboring a T528M or R278L mutation) or dark red circles (patients without T528M or 278L mutation), respectively. (C) Patients were grouped whether or not they have at least one allele of the c.1583C > T or c.833G > T mutation in their *PANK2* gene (T528M/R278L positive or negative). Mean values and standard error (SE) of the indicated parameters within these subgroups are given in percent of control (activity see also Table 2) or absolute numbers (BAD score and BAD score / yr, see also Table 1). An unpaired student’s *t*‐test was applied to assess the significance (*P*‐value) of the difference between respective means.

Together, in contrast to the surface mutations T528M and R278L, the L425P, D457Y, G521R and the E554K mutations are likely associated with major structural and/or functional impairment. Correspondingly, patients with the R278L or the T528M mutation in at least one of their *PANK2* alleles had residual PANK2 activities >10% (Fig. 3, Table 2). For further analyzes, we segregated our cohort of patients in a T528M/R278L‐positive (P8‐P14) and T528M/R278L‐negative (P1‐P7) group. These groups have nearly identical age at onset but significantly different means in PANK2 activity (Figs. 3 and 6C).

To evaluate the clinical relevance of these analyses, we assessed the link between PANK2 activity and the BAD score of the patients or an age‐normalized version thereof (BAD score / yr). PANK2 activity correlated with both the BAD score and the BAD score / yr with a Pearson’s correlation coefficient of −0.473 (*P* = 0.088) and −0.520 (*P* = 0.057), respectively. Interestingly, the BAD score and BAD score / yr correlated better with residual PANK2 activity in erythrocytes than with the age of onset (−0.166 (*P* = 0.571) and −0.408 (*P* = 0.147)) (Figs. 3 and 6A and B). Moreover, the means of the BAD score and of the BAD score / yr were lower in the group of T528M/R278L positive patients (Fig. 6C). Thus, erythrocyte PANK2 activity may be a predictor for the progression of neurodegeneration in PKAN.

Finally, we addressed whether T528M PANK2 mutants with residual activity remained responsive to physiologic regulation. Erythrocyte cytosol from a second blood donation of patients P9 and P10 (both T528M/G521R) revealed residual PANK2 activities of 11% and 12%, respectively. Similar to control (HC3), these activities were completely blocked by 5*µ*M acetyl‐CoA but increased by palmitoylcarnitine (data not shown). Thus, the T528M PANK2 mutant is accessible to metabolic regulators.

## Discussion

Here we show that PANK activity in erythrocytes is mediated by PANK2, assess the activity of PANK2 mutants in PKAN erythrocytes and suggest that erythrocyte PANK2 could serve as a predictor for the progression of PKAN neurodegeneration.

CoA is a known co‐factor for maintaining plasma membrane lipid metabolism in erythrocytes.^24^ Hence CoA biosynthesis is likely essential for erythrocyte homeostasis. This study provides evidence that it is the PANK isoform 2 that is required for CoA biosynthesis in erythrocytes: (1) The characteristics of PANK activation and inhibition by palmitoylcarnitine and acetyl‐CoA, respectively, are in line with the known features of the PANK2 isoform (Fig. 1).^13^, ^14^ (2) Western blot analysis of erythrocyte cytosolic extracts reveals the characteristic 49 kDa band of the mature PANK2 protein (Fig. 1C). (3) Most significantly, erythrocytes of PKAN patients have strongly reduced or absent PANK activity (Figs. 1D and 2, Table 2). In line with proteomic data,^22^, ^23^ this corroborates that PANK2 is the only PANK isoform in erythrocytes.

Mitochondrial localization and processing of PANK2 was described previously.^10^, ^11^ Mature PANK2 (49 kDa) is generated by two proteolytic events on the full‐length translation product (63 kDa) by the mitochondrial processing peptidase (MPP). It is puzzling that erythrocytes contain PANK2, since these cells lose mitochondria during terminal erythropoiesis. Two scenarios can be envisaged: (1) PANK2 is leaking from mitochondria to the cytosolic compartment during erythrocyte maturation. (2) An alternative in‐frame start AUG site (Met‐124) generates a cytosolic version of the PANK2 protein (Uniprot database as Q9BZ23‐3). In line with this scenario, dual targeting of proteins to mitochondria and the cytosolic compartment has been described.^25^ While we have no proof yet for the presence of this PANK2 sequence variant in the erythrocytes, the possibility that in neurons both a mitochondrial and a cytosolic version of PANK2 may be generated from the same mRNA transcript, has implications for the interpretation of PKAN pathology. CoA biosynthesis can take place independently at various subcellular locations within the topologically distinct cytosolic compartments of neurons. Subcellular CoA biosynthesis could be specifically regulated by local metabolic constraints necessitating the non‐redundant properties of cytosolic PANK1, PANK3, and PANK2 regarding their specific modes of activation by CoA metabolites. Thus, not only mitochondrial but also cytosolic PANK2‐specific aberrations may be causative factors for PKAN neurodegeneration.

We show that PANK2 activity in the erythrocytes of the patients negatively correlates with the severity of dystonia as estimated by an age‐normalized BAD score (BAD score / yr in Fig. 6B and C). In our cohort, this correlation is stronger than that between the age of onset and the severity of dystonia. Thus, early determination of erythrocyte PANK activity at the onset of the disease might be a prognostic marker for the progression of neurodegeneration. To confirm this, a prospective study with repetitive evaluation of BAD scores will be necessary. Patient P5 (G521R/D457Y) and patients P2 and P7 (P288R/S191fs) show the atypical form of PKAN and relatively low age‐normalized BAD scores despite of a (near) total lack of PANK2 activity in their erythrocytes (Fig. 6A and B). Possibly, the P288R and the D457Y mutants have higher stability and specific activity in neurons as compared to erythrocytes. Neuron‐specific chaperones,^26^ absent in erythrocytes, may well account for a rescue of activity and stability of these mutants in neurons and confer slower disease progression.

Our study reveals the potential of erythrocytes as patient‐derived cell system to study molecular aberrations in PKAN pathology. Being easily available, erythrocytes could turn out to be useful in studies on the cellular effects of novel PKAN drugs.^27^, ^28^ More generally, since recent analyses revealed a yet unrecognized complexity of the erythrocyte proteome and metabolome, erythrocytes may also be a valuable source for studying other disease‐causing mutations and/or pathological molecular mechanisms of congenital diseases. Lacking transcriptional and translational adaptation mechanisms, erythrocytes maintain their protein‐ and lipid‐based regulatory network and homeostatic features for considerable times after removal from the blood circuit so that proper analyses can unravel donor‐specific characteristics and even lifestyle‐ and diet‐dependent intra‐donor variations (unpublished observations). Thus, erythrocytes may be used as reporters on molecular effects of treatments and/or alimentation. Sophisticated tests on erythrocytes as a whole and/or analyses of erythrocyte‐derived samples may generate patient‐specific information with diagnostic and prognostic potential and thereby emerge as a new methodological approach in the field of personalized medicine not only in the context of rare congenital diseases.

## Conflict of Interest

Thomas Klopstock reports the following potential competing interests: served as coordinating investigator of the FORT trial; received research funding from Retrophin, Inc.; served as coordinating investigator of the deferiprone in PKAN randomized and extension trial; received research funding from ApoPharma Inc.; received support from the European Commission 7th Framework Programme (FP7/2007‐2013, HEALTH‐F2‐2011; grant agreement No. 277984, TIRCON) and from the European Reference Network for Rare Neurological Diseases (ERN‐RND), co‐funded by the European Commission (ERN‐RND: 3HP 767231); provides consulting services to ApoPharma Inc., CoA Therapeutics, TM3 Therapeutics, and Retrophin, Inc.; received travel support from ApoPharma Inc. The other authors report no potential competing interests.

## Author Contribution

MW, EWM, TK, and US contributed to the conception and design of the study. MW, EWM, GM, VD, KD‐C, DMB, BB, TK, and US contributed to the acquisition and analysis of data. MW, EWM, GM, HP, BB, TK, and US contributed to drafting the text and preparing the figures.

## References

[1] Zhou B , Westaway SK , Levinson B , et al. A novel pantothenate kinase gene (PANK2) is defective in Hallervorden‐Spatz syndrome. Nat Genet 2001;28:345–349. 11479594 10.1038/ng572

[2] Kurian MA , Hayflick SJ . Pantothenate kinase‐associated neurodegeneration (PKAN) and PLA2G6‐associated neurodegeneration (PLAN): review of two major neurodegeneration with brain iron accumulation (NBIA) phenotypes. Int Rev Neurobiol 2013;110:49–71. 24209433 10.1016/B978-0-12-410502-7.00003-X PMC6059649

[3] Prohaska R , Sibon OCM , Rudnicki DD , et al. Brain, blood, and iron: perspectives on the roles of erythrocytes and iron in neurodegeneration. Neurobiol Dis 2012;46:607–624. 22426390 10.1016/j.nbd.2012.03.006 PMC3352961

[4] Siegl C , Hamminger P , Jank H , et al. Alterations of red cell membrane properties in neuroacanthocytosis. PLoS One 2013;8:e76715. 24098554 10.1371/journal.pone.0076715 PMC3789665

[5] Schiessl‐Weyer J , Roa P , Laccone F , et al. Acanthocytosis and the c.680 A>G mutation in the PANK2 gene: a study enrolling a cohort of PKAN patients from the dominican, Republic. PloS One 2015;10:e0125861. 25915509 10.1371/journal.pone.0125861 PMC4411072

[6] Lupo F , Tibaldi E , Matte A , et al. A new molecular link between defective autophagy and erythroid abnormalities in chorea‐acanthocytosis. Blood 2016;128:2976–2987. 27742708 10.1182/blood-2016-07-727321 PMC5179337

[7] De Franceschi L , Bosman GJ , Mohandas N . Abnormal red cell features associated with hereditary neurodegenerative disorders: the neuroacanthocytosis syndromes. Curr Opin Hematol 2014;21:201–209. 24626044 10.1097/MOH.0000000000000035

[8] De Franceschi L , Tomelleri C , Matte A , et al. Erythrocyte membrane changes of chorea‐acanthocytosis are the result of altered Lyn kinase activity. Blood 2011;118:5652–5663. 21951684 10.1182/blood-2011-05-355339 PMC3217364

[9] Oberwagner W , Sauer T , Hermann A , et al. Drug‐induced endovesiculation of erythrocytes is modulated by the dynamics in the cytoskeleton/membrane interaction. Blood Cells Mol Dis 2017;07:15–22. 10.1016/j.bcmd.2017.03.004 28301811

[10] Hortnagel K , Prokisch H , Meitinger T . An isoform of hPANK2, deficient in pantothenate kinase‐associated neurodegeneration, localizes to mitochondria. Hum Mol Genet 2003;12:321–327. 12554685 10.1093/hmg/ddg026

[11] Kotzbauer PT , Truax AC , Trojanowski JQ , Lee VM . Altered neuronal mitochondrial coenzyme A synthesis in neurodegeneration with brain iron accumulation caused by abnormal processing, stability, and catalytic activity of mutant pantothenate kinase 2. J Neurosci 2005;25:689–698. 15659606 10.1523/JNEUROSCI.4265-04.2005 PMC6725318

[12] Alfonso‐Pecchio A , Garcia M , Leonardi R , Jackowski S . Compartmentalization of mammalian pantothenate kinases. PLoS One 2012;7:e49509. 23152917 10.1371/journal.pone.0049509 PMC3496714

[13] Zhang YM , Rock CO , Jackowski S . Biochemical properties of human pantothenate kinase 2 isoforms and mutations linked to pantothenate kinase‐associated neurodegeneration. J Biol Chem 2006;281:107–114. 16272150 10.1074/jbc.M508825200

[14] Leonardi R , Rock CO , Jackowski S , Zhang YM . Activation of human mitochondrial pantothenate kinase 2 by palmitoylcarnitine. Proc Natl Acad Sci USA 2007;104:1494–1499. 17242360 10.1073/pnas.0607621104 PMC1785270

[15] Zhang YM , Rock CO , Jackowski S . Feedback regulation of murine pantothenate kinase 3 by coenzyme A and coenzyme A thioesters. J Biol Chem 2005;280:32594–32601. 16040613 10.1074/jbc.M506275200

[16] Hayflick SJ , Westaway SK , Levinson B , et al. Genetic, clinical, and radiographic delineation of Hallervorden‐Spatz syndrome. N Engl J Med 2003;348:33–40. 12510040 10.1056/NEJMoa020817

[17] Hartig MB , Hortnagel K , Garavaglia B , et al. Genotypic and phenotypic spectrum of PANK2 mutations in patients with neurodegeneration with brain iron accumulation. Ann Neurol 2006;59:248–256. 16437574 10.1002/ana.20771

[18] Hong BS , Yun MK , Zhang YM , et al. Prokaryotic type II and type III pantothenate kinases: the same monomer fold creates dimers with distinct catalytic properties. Structure 2006;14:1251–1261. 16905099 10.1016/j.str.2006.06.008

[19] Vallari DS , Jackowski S , Rock CO . Regulation of pantothenate kinase by coenzyme A and its thioesters. J Biol Chem 1987;262:2468–2471. 3029083

[20] Hong BS , Senisterra G , Rabeh WM , et al. Crystal structures of human pantothenate kinases. Insights into allosteric regulation and mutations linked to a neurodegeneration disorder. J Biol Chem 2007;282:27984–27993 17631502 10.1074/jbc.M701915200

[21] Spry C , Saliba KJ . The human malaria parasite Plasmodium falciparum is not dependent on host coenzyme A biosynthesis. J Biol Chem 2009;284:24904–24913. 19584050 10.1074/jbc.M109.025312 PMC2757193

[22] Bryk AH , Wisniewski JR . Quantitative analysis of human red blood cell proteome. J Proteome Res 2017;16:2752–2761. 28689405 10.1021/acs.jproteome.7b00025

[23] D'Alessandro A , Righetti PG , Zolla L . The red blood cell proteome and interactome: an update. J Proteome Res 2010;9:144–163. 19886704 10.1021/pr900831f

[24] Arduini A , Mancinelli G , Radatti GL , et al. Role of carnitine and carnitine palmitoyltransferase as integral components of the pathway for membrane phospholipid fatty acid turnover in intact human erythrocytes. J Biol Chem 1992;267:12673–12681. 1618773

[25] Yogev O , Pines O . Dual targeting of mitochondrial proteins: mechanism, regulation and function. Biochim Biophys Acta 2011;1808:1012–1020. 20637721 10.1016/j.bbamem.2010.07.004

[26] Gorenberg EL , Chandra SS . The role of co‐chaperones in synaptic proteostasis and neurodegenerative disease. Front Neurosci 2017;11:248. 28579939 10.3389/fnins.2017.00248 PMC5437171

[27] Jeong SY , Hogarth P , Placzek A , et al. 4'‐Phosphopantetheine corrects CoA, iron, and dopamine metabolic defects in mammalian models of PKAN. EMBO Mol Med 2019;11:e10489. 31660701 10.15252/emmm.201910489 PMC6895607

[28] Sharma LK , Subramanian C , Yun MK , et al. A therapeutic approach to pantothenate kinase associated neurodegeneration. Nat Commun 2018;9:4399. 30352999 10.1038/s41467-018-06703-2 PMC6199309

